# Proteomic Analysis of One-carbon Metabolism-related Marker in Liver of Rat Offspring*[Fn FN2]

**DOI:** 10.1074/mcp.M114.046888

**Published:** 2015-09-04

**Authors:** Young-Ah You, Ji Hye Lee, Eun Jin Kwon, Jae Young Yoo, Woo-Sung Kwon, Myung-Geol Pang, Young Ju Kim

**Affiliations:** From the ‡Medical Research Institute, School of Medicine, Ewha Womans University, Seoul 158–710, Korea;; §Department of Obstetrics and Gynecology, Ewha Womans University, Seoul, 158–710, Korea;; ¶Department of Animal Science and Technology, Chung-Ang University, Anseong, Gyeonggi-Do 456–756, Korea

## Abstract

Maternal food intake has a significant effect on the fetal environment, and an inadequate maternal diet may result in intrauterine growth restriction. Intrauterine growth restriction newborn rat pups nursed by normal diet-fed dams exhibited rapid catch-up growth, which plays a critical role in the risk for metabolic and cardiovascular disease in later life. Specifically, one-carbon metabolism in the liver plays a critical role in placental and fetal growth. Impaired functioning of one-carbon metabolism is associated with increased homocysteine levels. In this study, we applied a comprehensive proteomic approach to identify differential expression of proteins related to one-carbon metabolism in the livers of rat offspring as an effect of maternal food restriction during gestation. Data are available via ProteomeXchange with identifier PXD002578. We determined that betaine-homocysteine S-methyltransferase 1, methylenetetrahydrofolate dehydrogenase 1, and ATP synthase subunit beta mitochondrial (ATP5B) expression levels were significantly reduced in the livers of rat offspring exposed to maternal food restriction during gestation compared with in the offspring of rats fed a normal diet (*p* < 0.05). Moreover, the expression levels of betaine-homocysteine S-methyltransferase 1, methylenetetrahydrofolate dehydrogenase 1, and ATP synthase subunit beta mitochondrial were negatively correlated with serum homocysteine concentration in male offspring exposed to maternal food restriction during gestation and normal diet during lactation. However, in female offspring only expression levels of methylenetetrahydrofolate dehydrogenase 1 were negatively correlated with homocysteine concentration. This study shows that maternal food restriction during late gestation and normal diet during lactation lead to increased homocysteine concentration through disturbance of one-carbon metabolism in the livers of male offspring. This suggests that male offspring have an increased gender-specific susceptibility to disease in later life through fetal programming.

Maternal nutrient intake during gestation affects fetal growth through nutritional and hormonal interactions between the mother, the placenta, and the fetus in humans and animal models (1–3). Specifically, non-optimal fetal environments with maternal food restriction result in intrauterine growth restriction (IUGR)^1^ (4). IUGR newborns nursed by normal diet-fed dams exhibited rapid catch-up growth, which plays a critical role in the risk for metabolic and cardiovascular disease in later life (5–7).

Our research group reported previously that rat offspring from maternal 50% food restriction (FR) during gestation and maternal normal diet during lactation demonstrated catch-up growth, resulting in obese offspring with higher levels of plasma triglycerides and leptin, with gender differences (8). In addition, maternal food restriction in rats caused decreases in the mass and number of pancreatic beta cells in the first generation of female offspring leading to insulin resistance and gestational hyperglycemia (9, 10). Maternal protein restriction also induces hypertension and vascular dysfunction in adult female rat offspring (11, 12). In sheep, maternal nutrient restriction impairs renal function, increases the development of glomerulosclerosis, and enhances apoptosis in kidneys, while altering the expression of proteins involved in regulating inflammatory processes (13, 14). This increased susceptibility to disease is explained during fetal programming by links between nutrition and epigenetic mechanisms (15, 16).

In placental and fetal growth, one-carbon metabolism is tightly connected to the methionine and folate cycle and is an important metabolic function of the liver (17–19). It can change the availability of the common methyl donor, S-adenosylmethionine (AdoMet). In this pathway, the methyl donor influences homocysteine remethylation to methionine (19). Impaired functioning of one-carbon metabolism affects several molecular or protein alterations, which can compromise cellular homeostasis and also trigger the development of several pathological states in humans and experimental animals. Hyperhomocystenemia resulting from impaired one-carbon metabolism is a risk factor for certain cancers (20, 21), cardiovascular disease (22–25), neural tube defects (26, 27), and Alzheimer's disease (28). However, little is known about the changes associated with one-carbon metabolism in the livers of IUGR offspring.

In this study, the focus was to determine the differential protein profiles of the livers of rat offspring following maternal 50% food restriction during late gestation and normal diet during lactation. First, we analyzed protein expression related to one-carbon metabolism in relation to maternal food intake using two-dimensional electrophoresis (2-DE). Next, the 2-DE results were confirmed using Western blotting and used to construct a related signaling pathway. Finally, we analyzed the correlation between protein expression and serum homocysteine (Hcy) concentration to define impaired one-carbon metabolism.

## EXPERIMENTAL PROCEDURES

### 

#### 

##### Animal Experiments

Studies were approved by the Animal Research Committee of the School of Medicine at Ewha Womans University and were in accordance with the international guidelines for the care of laboratory animals.

Eight-week-old male and female Sprague-Dawley (S.D.) rats were purchased from Orient Bio (Seongnam, Kyunggi-do, Korea). Rats were housed in a temperature-controlled room with a 12-h light/dark cycle and given access to water and non-purified standard laboratory chow (supplemental Table S1) (Purina, Pyeongtaek, Korea). After a 1-week acclimation period rats were mated. Females were examined for the presence of a vaginal plug, which was regarded as day 1 of pregnancy. At day 10 of gestation, S.D. pregnant rats (*n* = 10) were divided into two groups and given an *ad libitum* (AdLib) or 50% food restriction (FR) diet to term. All nutritional components were equally reduced in the 50% FR diet. After birth, we divided the rats into four offspring groups: AdLib/AdLib (given a normal diet during the pregnancy and lactation periods), AdLib/50% FR group (given 50% FR during the lactation period), FR/AdLib (given 50% FR during the pregnancy period), and FR/FR (given 50% FR during the pregnancy and lactation periods).

Body weights were measured at each indicated time point (birth, 3 days, and 3 weeks). Three-week-old offspring were sacrificed by exsanguination under Zoletil (Virbac, Carros, France) anesthesia and livers were immediately isolated and stored at −80 °C. Samples from offspring (*n* = 9 males, 9 females/group) were randomly chosen.

##### Protein Extraction

Approximately 0.2 g of frozen liver were prepared in lysis buffer, containing 20 mm Tris, 15 0 mm NaCl, 1% Triton X-100, 1 mm EDTA, 1 mm EGTA, 1 mm PMSF, 1 mm β-glycerol phosphate, l mm NaF, 1 mm Na_3_VO_4_, and Protease Inhibitor Mixture^TM^ (Roche Molecular Biochemicals, Indiananpolis, IN) on ice. Each sample was sonicated for 30 s and centrifuged at 14,000 rpm for 30 min at 4 °C. The supernatant was collected and the protein concentration was determined using a BCA protein assay kit (Sigma, St. Louis, MO). Equal amounts of lysate, 250 μg and 40 μg, were used for the 2-DE and Western blot analyses, respectively.

##### 2-DE Analysis and Image Analysis

To minimize individual differences in liver function, liver tissue from different individuals from the control, AdLib/FR, FR/AdLib, and FR/FR groups was combined and then the 2-DE and image analyses were performed three times using different combined samples (29, 30). First dimension electrophoresis was performed using an IPGphor IEF apparatus and then the strips were focused at 100V-1 h, 200V-1 h, 500V-1 h, 1000V-1 h, 5000V-1.5 h, 8000V-1.5 h, and 8000–90,000V h. After iso-electrofocusing, the strips were equilibrated with equilibration buffer A containing 6 m urea, 75 mm Tris-HCl (pH 8.8), 30%(v/v) glycerol, 2% (w/v) SDS, 0.002% (w/v) bromphenol blue, and 2% (w/v) DTT for 15 min at room temperature (RT). The strips were equilibrated a second time with equilibration buffer B (equilibration buffer A with 2.5% (w/v) iodoacetamide (Sigma, St. Louis, MO) without DTT for 15 min at RT. Two-dimensional electrophoresis was carried out using 12.5% (w/v) SDS-PAGE gels with the strips at 100 V 1 h and 500 V until the bromphenol blue front began to migrate off the lower end of the gels. The proteins were loaded into different 2D-analysis gels for each group. The molecular weights ranged from 6.5 to 200 kDa and pH from 3–11. The gels were silver-stained for image analysis following the manufacturer's instructions (Amersham Biosciences Pharmacia Bio-tech). Then, the gels were scanned using a high-resolution GS-800 calibrated scanner (Bio-Rad, Hercules, CA). Detected spots were matched and analyzed using the PD Quest 8.0 software (Bio-Rad).

##### Protein Identification

The proteins were subjected to in-gel trypsin digestion. Excised gel spots were destained with 100 ml of destaining solution (30 mm potassium ferricyanide, 100 mm sodium thiosulfate) while shaking for 5 min. After removal of the solution, the gel spots were incubated with 200 mm ammonium bicarbonate for 20 min. The gel pieces were dehydrated with 100 μl of acetonitrile and dried in a vacuum centrifuge. The above procedure was repeated three times. The dried gel pieces were rehydrated with 20 ml of 50 mm ammonium bicarbonate containing 0.2 mg modified trypsin (Promega, Madison, WI) for 45 min on ice. After removing the solution, 30 ml of 50 mm ammonium bicarbonate were added. The digestion was performed overnight at 37 °C. The peptide solution was desalted using a C18 nano column (Homemade).

Custom-made chromatographic columns were used to desalt and concentrate the peptide mixture prior to mass spectrophotometric analysis. A column consisting of 100–300 nl of Poros reverse phase R2 material (20–30 μm bead size, Perseptive Biosystems) was packed in a constricted GELoader tip (Eppendorf, Hamburg, Germany).

A 10-ml syringe was used to force liquid though the column by applying gentle air pressure. Thirty microliters of the peptide mixture from the digestion supernatant were diluted in 30 μl of 5% formic acid, loaded onto the column, and washed with 30 μl of 5%formic acid. For analysis by MS/MS, the peptides were eluted with 1.5 μl 50% ethanol/49% H_2_O/1% formic acid directly into a pre-coated borosilicate nano-electrospray needle (Hanger Worldwide Inc., Hickory, NC).

##### Electrospray Ionization MS/MS Analysis

MS/MS of the peptides generated by in-gel digestion was performed by nano-electrospray ionization (ESI) using a MicroQ-TOF2 III mass spectrometer (Bruker Daltonics, Bremen, Germany). The source temperature was room temperature (RT). A potential of 1 kV was applied to the precoated borosilicate nano-electrospray needles (Hanger Worldwide Inc) in the ion source, combined with a nitrogen back-pressure of 0–5 psi to produce a stable flow rate (10–30 μl/min). The cone voltage was 40 V. The quadruple analyzer was used to select precursor ions for fragmentation in the hexapole collision cell. The collision gas was argon at a pressure of 6–7 × 10^−5^ mbar and the collision energy was 25–40V.

##### Database Searching

MS/MS was assigned as the ion search option in MASCOT software (v2.4, Matrix Science, London, UK). Peptide fragment files were obtained from the peptide peaks in ESI-MS by ESI-MS/MS. Trypsin was selected as the enzyme with one potentially missing cleavage site. ESI-QTOF was selected as the instrument type. The peptide fragment files were searched based on the database by using the MASCOT search engine [Mascot search engine, Database: NCBInr 20140807 (47,520,513 sequences; 16,962,606,718 residues) Timestamp: 7 Jul 2015] and limited *to Rattus norvegicus*. Oxidized methionine was set as a variable modification, and carbamido methylated cysteine was set as a fixed modification. The mass tolerance was set at ± 0.5 and ± 0.8 Da for the peptides and fragments, respectively. High scoring peptides corresponded to peptides that were above the default significance threshold in MASCOT (*p* < 0.05, peptide score ≥ 35). The mass spectrometry proteomics data have been deposited to the ProteomeXchange Consortium (31) via the PRIDE partner repository with the data set identifier PXD002578.

##### Western Blot Analysis

To confirm the 2-DE results, Western blot analysis was conducted using the livers of 3-week-old offspring from the AdLib/AdLib (*n* = 4 males, *n* = 4 females), AdLib/FR, FR/AdLib, and FR/FR groups (*n* = 5 males, *n* = 5 females/group). Briefly, equal amounts of lysate (40 μg) were separated by SDS-PAGE and transferred to polyvinylidene difluoride membranes (Amersham Biosciences, Piscataway, NJ) at 80 V for 2 h. The membrane was blocked for 1 h in 2.5% skim milk in Tris-buffered saline (TBS) with 0.01% Tween-20 (TBS-T). Next, the membrane was washed in TBS-T and incubated with antimethylenetetrahydrofolate dehydrogenase 1 (1:1000; GeneTex Inc., Irvine, CA), antibetaine-homocysteine-S-methyltransferase (1:1000; Santa Cruz Biotechnology, Dallas, TX), anti-ATP synthase subunit beta (1:2000; GeneTex Inc.), and anti-αtubulin (1:2000; AbFrontier, Seoul, Korea) antibodies overnight at 4 °C. The membrane was washed in TBS-T. Secondary anti-rabbit or anti-mouse HRP-conjugate (Santa Cruz Biotechnology) was then added at 1:3000 dilution in blocking buffer for 1 h at room temperature. The membranes were then washed again in TBS-T, and developed using enhanced chemiluminescence reagents (Santa Cruz Biotechnology).

##### Signaling Pathway

To visualize the biological functions and signaling pathways of the differentially expressed proteins, the Pathway Studio software (v 9.0, Aridane Genomics) was used.

##### Determination of Homocysteine Concentration in Serum

The relationships between proteins confirmed by Western blot analysis and one-carbon metabolism were determined using serum Hcy concentration. Serum Hcy concentrations from 3-week-old offspring from the AdLib/AdLib and FR/AdLib (*n* = 9 males, *n* = 9 females/group) groups were measured by enzyme-linked immunoassay (ELISA) following the protocol in the Rat Homocysteine ELISA Kit (Cusabio Biotech Co. LTD, China).

##### Statistical Analysis

Statistical analyses were performed using SPSS version 18.0 (SPSS Inc., Chicago, IL). We used Student's *t* test (*p* < 0.05) to determine which protein spots were differentially abundant (> 10-fold) between the livers of 3-week-old offspring from the AdLib/AdLib and FR groups. The data are expressed as the means ± standard deviation (S.D.). We used the Wilcoxon rank-sum test to evaluate individual differences in two groups (*p* < 0.05). Spearman's correlations were calculated to analyze differences between the expressed proteins and serum Hcy concentration.

## RESULTS

### 

#### 

##### Changes in Body and Liver Weights of 3-week-old Offspring

Maternal food restriction during late gestation resulted in low birth weight (*p* < 0.01, Table I). After 3 days, the offspring of dams administered a FR diet during late gestation weighed significantly less than did those of AdLib dams (*p* < 0.01, Table I). However, although the body and liver weights of the IUGR offspring were lower than those of the AdLib offspring at birth, the 3-week-old IUGR offspring nursed by AdLib dams showed a marked increase in body and liver weights that exceeded those of the AdLib/AdLib offspring. In contrast, the body weight of offspring nursed by FR dams was significantly lower than that of AdLib/AdLib offspring at 3 weeks (*p* < 0.05).

**Table I:** Comparison of body and liver weight at 3 day and 3weeks after birth

		Ad lib*^a^*/Ad lib*^b^*	Ad lib/FR	FR/Ad lib	FR/FR
Body weight (g)	Birth	7.63 ± 0.32		6.92 ± 0.81**	
	3 days	9.69 ± 0.63	8.44 ± 0.78**	8.14 ± 0.86**	7.56 ± 0.72**
	3 weeks	56.30 ± 3.66	18.10 ± 1.19**	54.58 ± 8.50	16.67 ± 1.70**
Liver wet weight (g)	3 days	0.28 ± 0.04	0.22 ± 0.05*	0.29 ± 0.06	0.21 ± 0.05*
	3 weeks	2.36 ± 0.39	0.52 ± 0.09**	1.98 ± 0.38	0.49 ± 0.08**

*^a^* Maternal diet from 10 day of pregnancy to delivery.

*^b^* Maternal diet on lactation period for 3 weeks.

* is significant difference compared to AdLib/AdLib (*p* < 0.05).

** is significant difference compared to AdLib/AdLib (*p* < 0.01).

##### Proteomic Analysis of Protein Expression in the Liver of 3-week-old Offspring

**Fig. 1.**
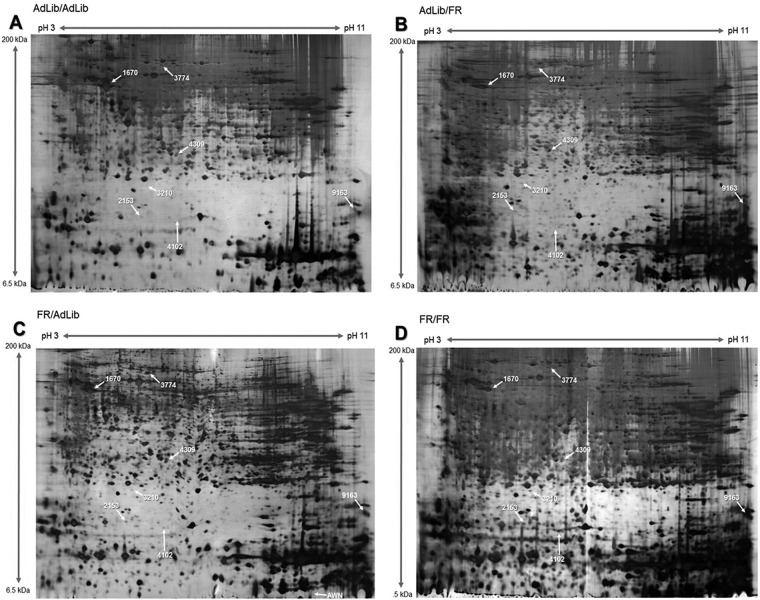
**Separation of proteins by 2-DE.** 2-DE gels were stained with silver nitrate and analyzed using the PDQuest 8.0 software. Protein spots from the livers of offspring from (*A*) AdLib/AdLib, (*B*) AdLib/FR, (*C*) FR/AdLib, and (*D*) FR/FR groups.

To analyze the effect of fetal programming on liver function, we compared the protein expression profiles in the livers of the 3-week-old offspring. We identified protein spots in the FR/AdLib offspring that exhibited a 10-fold difference in protein expression compared with the livers of AdLib/AdLib offspring (*p* < 0.05, Fig. 1). Table II shows the differential expression of liver proteins in the AdLib/AdLib control and experimental groups. The levels of expression of BHMT1, MTHFD1, and ATP5B were significantly reduced in the livers of FR/AdLib pups compared with those of the AdLib/AdLib offspring (*p* < 0.05). Moreover, the levels of expression of BHMT1, MTHFD1were significantly reduced (*p* < 0.05), but ATP5B was substantially elevated in the livers of FR/FR offspring.

**Table II:** Comparison of protein expression in liver of rat offspring by proteomics

Spot No.*^a^*	gi No. *^b^*	Protein description	Symbol	MS*^c^*	Fold change*^d^*		
					1) vs. 2)	1) vs. 3)	1) vs. 4)
1670	gi 6729935	ATP synthase subunit beta, mitochondrial	ATP5B	2105	0.56	0.10*	7.11
2153	gi 13624295	Glia maturation factor beta	GMFB	201	0.97	0.10	0.06
3210	gi 13540663	Betaine–homocysteine S-methyltransferase 1	BHMT1	84	0.37	NE*	NE*
3774	gi 929988	Pyruvate carboxylase	PC	634	1.90	0.35	4.74
4102	gi 4139571	Adenosylhomocysteinase	AHCY	195	0.40	0.07	NE
4309	gi 59808745	Methylenetetrahydrofolate dehydrogenase 1	MTHFD1	328	1.03	0.03*	0.31*
9163	gi 20302061	ATP synthase subunit O, mitochondrial precursor	ATP5O	104	1.91	0.09	0.20

*^a^* Spot number in the 2D-analysis.

*^b^* Protein accession number for NCBI database.

*^c^* MS: MASCOT score is −10 log (p), where p is the probability that the observed match is a random event. Scores >35 indicate identity or extensive homology (*p* < 0.05).

*^d^* is fold changes of proteins expressed in liver of offspring from 1); Ad Lib/Ad Lib group, 2); Ad Lib/ FR group, 3); FR/AdLib group and 4); FR/FR group. NE: Not expression.

* *p* < 0.05 compared with AdLib/AdLib.

##### Protein Confirmation Using Western Blotting

**Fig. 2.**
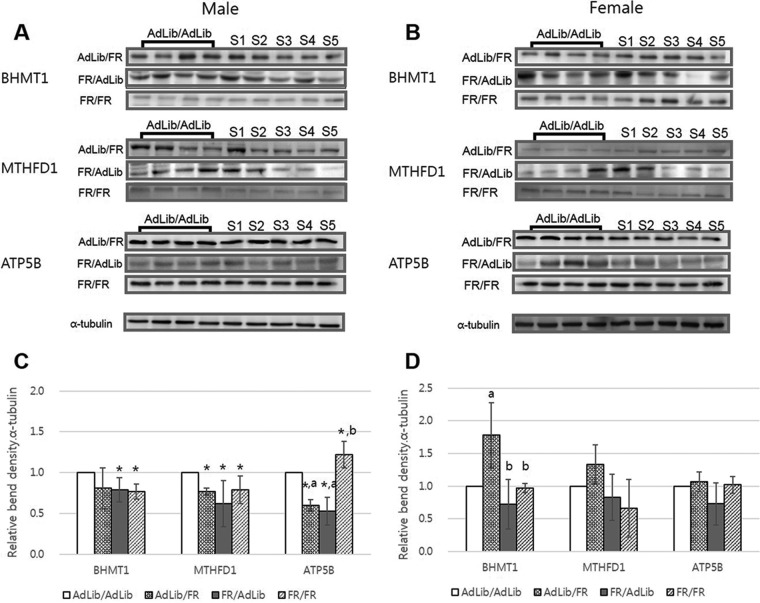
**Comparison of liver protein expression in the offspring according to maternal diet.** The ratio of BHMT1, MTHFD1, and ATP5B density (optical density [OD × mm]/α-tubulin [OD × mm]) in the livers of male (*A*) and female (*B*) offspring. The number of samples (S) signify the protein density of the offspring in each group. The levels of expression of BHMT1, MTHFD1, and ATP5B were significantly reduced in the livers of the FR/AdLib male offspring (*p* < 0.05, *C*) compared with those of the control group. The levels of BHMT1, MTHFD1, and ATP5B in the livers of female offspring did not differ among groups (*D*). *p < 0.05 compared with AdLib/AdLib; ^a, b^ significant difference of the protein expressions between same protein (*p* < 0.05). Data are expressed as means ± S.D.

The differential expression of proteins identified using 2-DE was further examined by Western blotting analysis using commercially available antibodies (Fig. 2). The Western blot revealed that the levels of expression of BHMT1, MTHFD1, and ATP5B were significantly reduced in the livers of the male offspring of FR/AdLib dams, which was consistent with the 2-DE findings (*p* < 0.05, Fig. 2*A* and 2*C*); however, the liver levels of BHMT1, MTHFD1, and ATP5B did not differ among female offspring (Fig. 2*B* and 2*D*). BHMT1 and MTHFD1 expression was significantly reduced in the livers of the FR/FR male offspring, whereas ATP5B expression in this group was significantly increased (*p* < 0.05, Fig 2*A* and 2*C*). We found no difference in the expression of these proteins in the liver of the FR/FR female offspring (Fig. 2*B* and 2*D*).

##### Signaling Pathway

The gene name of each differentially expressed protein identified via a database search was imported into Pathway Studio to determine the related signaling pathways. We included all proteins described in Table III in construction of the signaling pathways.

**Table III:** Signaling pathways associated with differentially expressed proteins as identified by Pathway Studio. Differentially expressed proteins were entered into Pathway Studio to identify the signaling pathways. Among the differentially expressed proteins (> 10-fold) in liver of offspring from Ad lib/Ad lib and FR/Ad lib groups, at least 5 exhibited regulatory roles in single or more pathways simultaneously (*p* < 0.05).

Signaling Pathways	Overlapping Entities	*p* value
Ariadne Metabolic Pathways		
Methionine metabolism	Bhmt1, Ahcy	0.0004
Respiratory chain and oxidative phosphorylation	Atp5b, ATP5O	0.0220

**Fig. 3.**
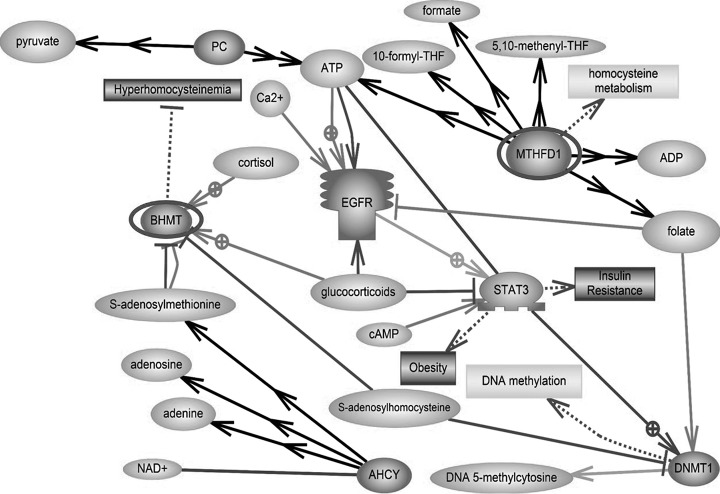
**Signaling pathways.** The schematic was developed using Pathway Studio 9.0 following a database search in PubMed. Red-highlighted proteins were more highly expressed in the livers of offspring of the FR/Ad Lib group than the AdLib/AdLib group (*p* < 0.05). PC, pyruvate carboxylase, mitochondrial precursor; BHMT, betaine-homocysteine S-methyltransferase 1; AHCY, adenosylhomocysteinase; MTHFD1, methylenetetrahydrofolate dehydrogenase 1; EGFR, epidermal growth factor receptor; STAT3, signal transducers and activators of transcription 3; DNMT1, DNA methyltransferase 1.

Two pathways were significantly correlated with four proteins (*p* < 0.05, Table III). BHMT1 and adenosylhomocysteinase (Ahcy) were related to methionine metabolism. ATP5B and ATP5O were related to the respiratory chain and oxidative phosphorylation. The schematic image includes a link to both the physiological function and interactions of identified proteins. The discovered proteins interact with each other. Moreover, we focused on hyperhomocystenemia related to impaired folate-methionine metabolism (Fig. 3).

##### The Relationship Between Protein Expression and Homocysteine Concentration

**Fig. 4.**
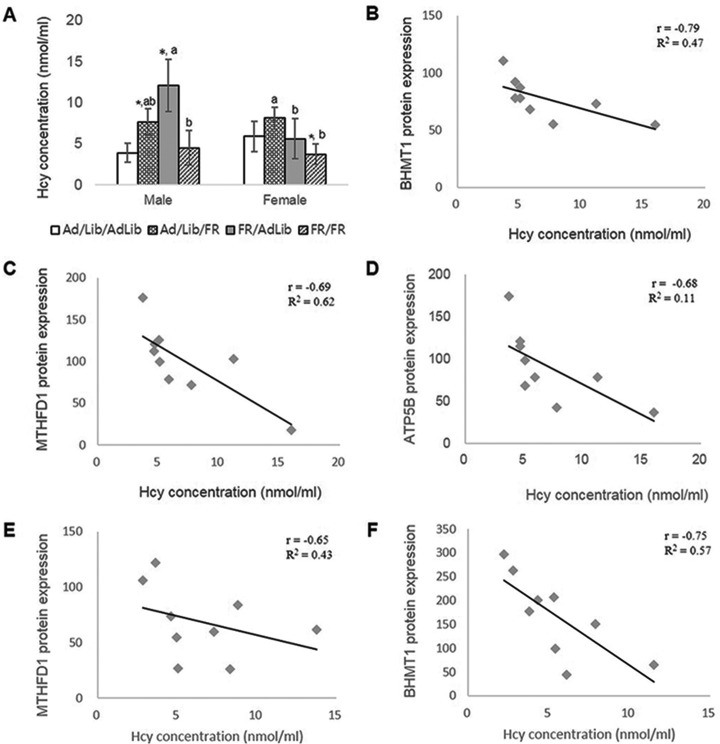
**The relationship between protein expression and serum Hcy concentration in the liver of offspring.** Comparison of serum Hcy concentrations in all offspring (*A*). The correlation between levels of protein expression and Hcy concentrations. BHMT1 (*B*), MTHFD1 (*C*), and ATP5B (*D*) in FR/AdLib male offspring (*n* = 9). MTHFD1 in FR/AdLib female offspring (E, *n* = 9). BHMT1 in FR/FR female offspring (F, *n* = 9) *p < 0.05 compared with AdLib/AdLib; ^a, b^significant difference of Hcy concentrations in offspring according to sex (*p* < 0.05).

We used serum Hcy concentration to indicate impaired one-carbon metabolism in 3-week-old offspring. Maternal diet during late gestation and/or lactation was associated with significant changes in serum Hcy concentrations among pups (Fig 4*A*). Notably, the serum Hcy concentrations in the male offspring of AdLib/FR and FR/AdLib dams were significantly higher than those of the male offspring of AdLib/AdLib dams (*p* < 0.05), whereas the serum Hcy concentrations did not differ between the FR/FR and AdLib/AdLib male offspring. In female offspring, the levels of Hcy did not differ among the AdLib/FR, FR/AdLib, and control groups; however, the Hcy concentrations of FR/FR offspring were significantly lower than them (*p* < 0.05, Fig 4*A*). The Hcy concentration of AdLib/FR offspring were significantly higher than those of the FR/AdLib and FR/FR pups (*p* < 0.05).

Levels of BHMT1, MTHFD1, and ATP5B expression were negatively correlated with Hcy concentrations in the livers of male FR/AdLib offspring (*r* = −0.79, −0.69, and −0.68, respectively; *p* < 0.05, Fig. 4*B*, 4*C*, and 4*D*). In the female FR/AdLib offspring, MTHFD1 expression in the liver was negatively correlated with Hcy concentration (*r* = −0.75 *p* < 0.05; Fig. 4*E*). Furthermore, levels of BHMT1 expression were negatively correlated with Hcy concentrations in the livers of the FR/FR female offspring (*r* = −0.750 *p* < 0.05; Fig. 4*E*), but not in the offspring of the other dietary groups. Thus, compared with the AdLib/AdLib male offspring, the increase in Hcy levels and the negative correlation with protein expression were greater in the FR/AdLib male offspring than in males in the other groups (*p* < 0.05).

## DISCUSSION

In this study, we used a comparative proteomic analysis to demonstrate that maternal food intake during late gestation and lactation was associated with an alteration in hepatic growth and one-carbon metabolism. Our study is the first to identify alterations in BHMT1, MTHFD1, and ATP5B as fetal programming-related impaired one-carbon markers. Interestingly, these proteins were negatively correlated with the elevation of serum Hcy concentrations in FR/AdLib male offspring. These findings suggest that the male offspring of FR/AdLib dams may be at risk of increased susceptibility to diseases in later life.

Several studies in humans and animals have suggested a correlation between maternal food intake, birth weight, and susceptibility to diseases such as obesity, type 2 diabetes, and hypertension in later life (5–7, 32). Previous experimental animal models using maternal food restriction during gestation and maternal normal diet during lactation have confirmed the association between low birthweight and metabolic syndrome (33–35). A protein-restricted diet in pregnant rats has been associated with the risk of hypertension and renal deficits in later life (11). Desai and colleagues reported that IUGR offspring nursed by AdLib dams were obese as adults and showed evidence of hyperglycemia and insulin resistance (36).

Subsequent studies have shown that changes in the expression of a number of genes are associated with the metabolism of specific organs, such as the placenta, liver, kidney, and hypothalamus (37, 38). Rees and colleagues (39) reported that maternal protein restriction resulted in hypermethylation of DNA in the fetal liver. Recently, we reported reduced expression of ubiquitin carboxy-terminal hydrolase L1 in the brains of IUGR offspring associated with obesity (40). Thus, the postnatal growth of IUGR newborns nursed by dams fed a normal diet during lactation can be influenced by immediate and long-term programming.

It is apparent from the differential levels of expression of BHMT1, MTHFD1, and ATP5B that these proteins are involved in methionine metabolism and respiratory chain and oxidative phosphorylation in the livers of offspring (Table II). A protein-deficient maternal diet has been shown to disrupt this pathway and alter Hcy concentrations (41). Interestingly, although the liver weights and phenotypes of FR/AdLib male offspring were similar to those of the control pups at 3 weeks of age, the levels of expression of MTHFD1, BHMT1, and ATP5B were lower and the serum Hcy concentrations were higher in the FR/AdLib male offspring compared with the controls. In contrast, although we found a tendency toward increased expression of these proteins in female offspring compared with controls, the difference did not reach statistical significance.

Elevated plasma Hcy concentrations are associated with impaired one-carbon metabolism resulting from protein malnutrition and deficiencies in folate and vitamin B12 (42). Rees (43) proposed that the methionine content in a protein-restricted diet exceeded the nutritional requirement, resulting in elevated maternal serum Hcy. We showed that serum Hcy was increased in the 3-week-old male offspring of dams fed a 50% FR/AdLib diet, suggesting that differential food intake during late gestation and lactation affected the capacity for remethylation of homocysteine to methionine in the livers of the offspring. The increased Hcy concentration can be converted to cysteine via the trans-sulfuration pathways or recycled to methionine. Thus, changes in the plasma concentration of Hcy are related to alterations in cellular metabolism leading to Hcy accumulation in the cell (44). Thus, the increase in serum Hcy concentration may be the result of impaired one-carbon metabolism through the decreased expression of the BHMT 1 and MTHFD1 proteins in the livers of offspring.

BHMT and MTHFD1 activity is required to maintain adequate levels of liver AdoMet and total plasma Hcy (41). BHMT resides at the interface between choline oxidation, sulfur amino acid and one-carbon metabolism (45). Activity levels of hepatic BHMT are dose-dependently affected by methionine and methyl donors in the diet, including choline and betaine (46, 47). In addition, BHMT activity is required to maintain normal liver glutathione levels and prevent an accumulation of liver fat under certain dietary conditions (48). MTHFD1 is a C1-tetrahydrofolate (THF) synthase, which interconverts folate derivatives between various oxidation states required by thymidine or purine synthesis (49). MTHFD1 is critical for normal cellular function, growth, and differentiation, and MTHFD catalyzes the NADPH-dependent and reversible reduction of 5,10-methenyl-THF to 5,10-methylene-THF (50).

Decreased levels of expression of BHMT and MTHFD1 lead to an increase in serum Hcy concentrations. Furthermore, BHMT may cause the accumulation of hepatic fat through increased visceral fat and serum triglyceride levels (TG) (51). We previously reported that serum TG levels were substantially elevated in 3-week-old FR/AdLib offspring; at 24 weeks of age, the serum TG levels were significantly elevated compared with those of other groups (8). Desai reported that serum TG levels were significantly elevated in 3- and 36-week-old FR/AdLib offspring (36). Disruption of MTHFD1 in mice was associated with a reduction in hepatic AdoMet levels, which is a source of one-carbons for cellular methylation reactions (52). Moreover, alterations in the MTHFD1 enzyme have been shown to increase serum Hcy concentrations and the risk of folate-sensitive neural tube defects (53–55).

Our findings indicate that ATP5B (mitochondrial ATP synthase) levels were reduced in the FR/AdLib offspring and elevated in the FR/FR offspring. Hepatic ATPase related to the production and oxidation of fatty acid has been shown to be associated with the export of TG in the liver (51, 56), suggesting that reduced ATP5B levels in the FR/AdLib offspring may be involved in the accumulation of hepatic fat. Although the expression of BHMT1 and MTHFD1 was reduced in the male offspring of FR/AdLib and FR/FR dams compared with that of the AdLib/AdLib offspring, ATP5B expression was lower in the FR/AdLib and higher in the FR/FR offspring than in the AdLib/AdLib control pups. This finding suggests that the high levels of ATP5B in the livers of the FR/FR offspring acted to normalize homocysteine levels, whereas reduced levels of expression of ATP5B in the livers of FR/AdLib offspring played a role in the elevated levels of Hcy. Thus, fetal programming in which dams were fed a FR diet during late gestation and normal diet during lactation impaired liver function in their offspring.

Taken together, our findings suggest that differential food intake by dams during late gestation and lactation has a significant effect on the expression of BHMT1, MTHFD1, and ATP5B in the liver of 3-week-old offspring. In particular, the expressions of BHMT1 and MTHFD1were associated with significant increases in serum Hcy concentrations in male offspring through the disturbance of one-carbon metabolism. This suggests that maternal food restriction during late gestation and a normal maternal diet during lactation lead to gender-specific defects in liver function related to one-carbon metabolism in rat offspring. Moreover, male offspring have an increased susceptibility to disease, such as metabolic syndrome, cardiovascular disease, and neural tube defects.

## Supplementary Material

Supplemental Data

## Footnotes

^1^ The abbreviations used are:

IUGR: intrauterine growth restriction
AdLib: a*d libitum*
AdoMet: S-adenosylmethionine
Ahcy: adenosylhomocysteinase
ATP5B: ATP synthase subunit beta, mitochondrial
ATP5O: ATP synthase subunit O, mitochondrial precursor
BHMT1: betaine-homocysteine S-methyltransferase 1
ELISA: enzyme-linked immunosorbent assay
FR: food restriction
Hcy: homocysteine
MTHFD1: methylenetetrahydrofolate dehydrogenase 1
THF: C_1_-tetrahydrofolate
SD: Sprague-Dawley rat.
