# Supplementary material for: Proteomic Analysis of One-carbon Metabolism-related Marker in Liver of Rat Offspring
Source: Mol Cell Proteomics. 2015 Sep 4;14(11):2901–9. doi: 10.1074/mcp.M114.046888 (PMC4638034; doi:10.1074/mcp.M114.046888)
Supplement: Supplemental Data [file supp_14_11_2901__index.html]

Proteomic analysis of one-carbon metabolism-related marker in liver of rat offspring — Proteomic Analysis of One-carbon Metabolism-related Marker in Liver of Rat Offspring — 2-DE Analysis of Hepatic One-carbon Metabolism — Supplemental Data 

# Proteomic Analysis of One-carbon Metabolism-related Marker in Liver of Rat Offspring

## Supplemental Data

- Supplemental Table 1.pdf - Supplemental Table 1. Chemical composition of Purina Lab. rodent chow
