# Supplementary material for: Proteomic Analysis of One-carbon Metabolism-related Marker in Liver of Rat Offspring
Source: Mol Cell Proteomics. 2015 Sep 4;14(11):2901–9. doi: 10.1074/mcp.M114.046888 (PMC4638034; doi:10.1074/mcp.M114.046888)
Supplement: Supplemental Data [file supp_M114.046888_mcp.M114.046888-1.pdf]

Supplemental Table 1. Chemical composition of Purina Lab. rodent chow

Nutrients		
**Protein**	**%**	**20.00**
Arginine	%	1.26
Cystine	%	0.37
Glycine	%	0.87
Histidine	%	0.50
Isoleucine	%	0.82
Leucine	%	1.47
Lysine	%	1.01
Methionine	%	0.33
Phenylalanine	%	0.98
Tyrosine	%	0.63
Threonine	%	0.72
Tryptophan	%	0.25
Valine	%	0.91
**Fat** (ether extract)		4.50
Linoleic Acid	%	1.10
Linolenic Acid	%	0.12
Arachidonic Acid	%	0.02
Omega-3 Fatty Acids	%	1.11
**Fiber (Crude)**	**%**	**6.00**
Minerals		
**Ash**	**%**	**7.25**
Calcium	%	1.20
Phosphorus	%	0.62
Phosphorus (non-phytate)	%	0.40
Potassium	%	0.82
Magnesium	%	0.16
Sulfur	%	0.22
Sodium	%	0.34
Chloride	%	0.47
Fluorine	ppm	21.38
Iron	ppm	112.93
Zinc	ppm	128.85
Manganese	ppm	95.49
Copper	ppm	22.74
Cobalt	ppm	0.76
Iodine	ppm	1.42
Chromium	ppm	0.00
Selenium	ppm	0.32
Vitamins		
Vitamin K	ppm	6.69
Thyamin Hydrichloride	ppm	11.02
Riboflavin	ppm	11.57
Niacin	ppm	217.70
Pantothenic Acid	ppm	88.72
Choline Chloride	ppm	3447.96
Folic Acid	ppm	13.60
Pyridoxine	ppm	11.00
Biotin	ppm	0.15
B12	ppm	41.00
Vitamin A	IU/g	28.03
Vitamin D3(added)	IU/g	4.00
Vitamin E	IU/Kg	100.00
Calories provided by:		
Protein	%	24.52
Fat (ether extract)	%	12.41
Carbohydrates	%	63.07

*Nutrients expressed as percent of ration except where otherwise indicated. Moisture content is assumed to be 10% for the purpose of calculations.
